# A Loop-mediated Isothermal Amplification (LAMP) Assay for Rapid Identification of *Bemisia tabaci*

**DOI:** 10.3791/58502

**Published:** 2018-10-29

**Authors:** Simon Blaser, Hanspeter Diem, Andreas von Felten, Morgan Gueuning, Michael Andreou, Neil Boonham, Jennifer Tomlinson, Pie Müller, Jürg Utzinger, Beatrice Frey, Jürg E. Frey, Andreas Bühlmann

**Affiliations:** ^1^Department of Method Development and Analytics, Agroscope; ^2^Swiss Tropical and Public Health Institute; ^3^University of Basel; ^4^Swiss Federal Plant Protection Service, Federal Office for Agriculture; ^5^OptiGene Limited; ^6^Fera Science Limited; ^7^School of Natural and Environmental Sciences, Newcastle University; ^8^Department of Plants and Plant Products, Agroscope

**Keywords:** Environmental Sciences, Issue 140, *Bemisia tabaci*, LAMP, loop-mediated isothermal amplification, point of entry diagnostics, plant health, rapid diagnostics, quarantine organisms

## Abstract

The whitefly *Bemisia tabaci* (Gennadius) is an invasive pest of considerable importance, affecting the production of vegetable and ornamental crops in many countries around the world. Severe yield losses are caused by direct feeding, and even more importantly, also by the transmission of more than 100 harmful plant pathogenic viruses. As for other invasive pests, increased international trade facilitates the dispersal of *B. tabaci* to areas beyond its native range. Inspections of plant import products at points of entry such as seaports and airports are, therefore, seen as an important prevention measure. However, this last line of defense against pest invasions is only effective if rapid identification methods for suspicious insect specimens are readily available. Because the morphological differentiation between the regulated *B. tabaci* and close relatives without quarantine status is difficult for non-taxonomists, a rapid molecular identification assay based on the loop-mediated isothermal amplification (LAMP) technology has been developed. This publication reports the detailed protocol of the novel assay describing rapid DNA extraction, set-up of the LAMP reaction, as well as interpretation of its read-out, which allows identifying *B. tabaci* specimens within one hour. Compared to existing protocols for the detection of specific *B. tabaci* biotypes, the developed method targets the whole *B. tabaci* species complex in one assay. Moreover the assay is designed to be applied on-site by plant health inspectors with minimal laboratory training directly at points of entry. Thorough validation performed under laboratory and on-site conditions demonstrates that the reported LAMP assay is a rapid and reliable identification tool, improving the management of *B. tabaci. *

**Figure:**
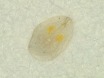


## Introduction

The whitefly *Bemisia tabaci* (Gennadius) is an invasive insect pest affecting the yield of many economically important crops including ornamental plants, vegetables, grain legumes, and cotton^1^,^2^. Beside damage caused through direct phloem-feeding, the homopteran species harms plants indirectly by the excretion of large amounts of honeydew onto the surfaces of leaves and fruits, as well as by the transmission of numerous plant pathogenic viruses^1^,^3^,^4^. Recent genetic studies comparing DNA sequences of the mitochondrial gene cytochrome *c* oxidase 1 (COI) revealed that *B. tabaci* is a species complex of at least 34 morphocryptic species^3^,^4^. Two highly invasive and damaging members within this complex, biotype B originating from the Middle East and the Asian Minor region, as well as biotype Q originating from the Mediterranean region, have been dispersed globally through international trading activities with plant products, particularly by the transportation of ornamentals^1^,^5^,^6^. Due to its worldwide pest status, the International Union for the Conservation of Nature and Natural Resources (IUCN) listed *B. tabaci* as one of the "world's 100 worst invasive alien species" and members of the species complex are regulated organisms by many countries^1^,^3^,^4^.

In the European Union (EU), *B. tabaci* is listed in the Plant Health Directive 2000/29/EC Annex 1AI as a quarantine organism whose introduction from non-EU countries and its dissemination within the EU are banned^4^. An essential prevention measure against the spread of quarantine organisms is the inspection of plant shipments at points of entry (POEs) such as airports and seaports^7^,^8^. In the case a quarantine organism is found, the National Plant Protection Organization (NPPO) in charge takes action by either rejecting or treatment (including destruction) of the infested shipment^9^. However, officers inspecting the imports often do not have the taxonomic expertise to accurately identify the vast range of pest species associated with global trade^9^. Especially the identification of immature life stages (*e.g., *eggs and larvae) without distinct morphological keys is virtually impossible for non-taxonomists^8^,^9^,^10^. Consequently, to enable implementation of quarantine measures with minimal delay, there is a need for alternative, rapid on-site identification assays^9^.

A candidate method is the loop-mediated isothermal DNA amplification (LAMP) technology that has recently been shown to be a suitable technology for the identification of plant pathogens^11^,^12^,^13^. LAMP is highly specific because the method uses at least two primer pairs recognizing six distinct DNA target sequences^14^. Due to the DNA strand displacement activity of the *Bst* DNA polymerase, LAMP reactions are performed under isothermal conditions^14^. Hence, in contrast to conventional polymerase chain reaction (PCR)-based assays there is no need for a thermal cycler^13^,^14^. Another advantage over PCR-based assays is its resilience against potential inhibitors in the DNA extract, circumventing the need for a DNA purification step^13^. Due to the protocol's speed and simplicity, LAMP may even be performed under on-site conditions using a portable, battery driven real-time detection device^8^,^15^.

A LAMP assay was designed in response to the demand for a rapid on-site identification method for *B. tabaci*^8^. The overarching aim was to develop a protocol that can be performed by plant health inspectors with limited laboratory training. A strong focus was, therefore, set on optimizing speed and simplicity of the protocol. While existing diagnostic tests have generally been developed for the identification of one or several biotypes of *B. tabaci*, the novel LAMP assay covers the whole *B. tabaci* species complex^8^,^16^,^17^,^18^. The problem of the pronounced genetic within-taxon diversity of the complex was solved by using combinations of different primer sets and the application of degenerate primers^8^. The novel *B. tabaci* LAMP assay is designed in such a way that the primers target a fragment at the 3' end of the mitochondrial COI gene^8^. This gene presents a suitable target for animal diagnostic assays because it harbors regions conserved enough to ensure diagnostic sensitivity for a specific species, while discriminating enough between closely related organisms^19^,^20^. Furthermore, the COI gene is often used as a genetic marker in population genetic studies and as a signature sequence in DNA barcoding analyses, resulting in numerous DNA sequence entries in open source databases such as GenBank and BOLD^21^,^22^. Beside the publicly available COI sequences from *B. tabaci*, COI sequences from closely related species (*Aleurocanthus* spp. [N = 2], *Aleurochiton aceris*, *Aleurodicus dugesii*, *Bemisia* spp. [N = 3], *Neomaskellia andropogonis*, *Tetraleurodes acaciae*, and *Trialeurodes* spp. [N = 4]) were included in the primer design of this study and used to assess diagnostic sensitivity and specificity *in silico*^8^.

Due to the accuracy of the method, its speed (<1 h) and the simplicity of the protocol, the assay has been shown to be suitable for on-site application when implemented as part of the import control procedure at a Swiss POE^8^.

## Protocol

### 1. Preparations

1. Preparing aliquots of alkaline DNA extraction solution.
  1. Produce a stock of alkaline DNA extraction solution using molecular grade water supplemented with 600 µM potassium hydroxide (KOH) and 2 µM Cresol Red. CAUTION: KOH is a strong base dissolved in water. Avoid spills, and skin and eye contact.
  2. Dispense 30 µL of alkaline DNA extraction solution (prepared in step 1.1.1) into 0.5 mL microcentrifuge tubes and store the aliquots at 4 °C. NOTE: Use the aliquoted DNA extraction solution within 1 year.
2. Preparing *B. tabaci* positive amplification control (PAC).
  1. Generate PCR amplicons of the LAMP target DNA fragment. NOTE: An introduction into general PCR principles and practices is given by Lorenz^23^.
    1. Synthesize or obtain the primers C1-J-2195 (5’-TTGATTTTTTGGTCATCCAGAAGT-3’) and TL2-N-3014 (5’-TCCAATGCACTAATCTGCCATATTA-3′) amplifying a fragment of the mitochondrial COI gene^24^,^25^.
    2. Set up the PCR reaction as described in **Table 1**. Use DNA extract (see step 2.1) of a reference *B. tabaci* specimen as DNA template. NOTE: Optionally, it is possible to extract the *B. tabaci* DNA for the PAC using a commercial kit according to the manufacturer’s instructions.
    3. Program a thermal cycler using the following conditions: 15 min at 95 °C; 45 cycles of 40 s at 95 °C, 15 s at 45 °C, ramping over 60 s to 60 °C, 2 min at 72 °C; 7 min at 72 °C; hold at 4 °C.
    4. Clean the PCR amplification product using a commercial PCR clean-up kit according to the manufacturer’s protocol and elute the final product in molecular grade water.
    5. Use a commercial kit with DNA-intercalating dye to measure the DNA concentration of the PCR amplification product according to the manufacturer’s instructions and dilute with molecular grade water to a concentration of 1 ng/µL. Store the diluted PCR amplification product as PAC stock solution at -20 °C. NOTE: Use the PAC stock solution within 1 year.
    6. Supplement the PAC stock solution (prepared in step 1.2.1.5) with 0.6 µM KOH and dilute with molecular grade water to a concentration of 5 x 10^-3^ ng/µL. Store the product at 4 °C. NOTE: Use the PAC within 5 h for the preparation of the ready-to-use *B. tabaci* LAMP kits described in the next step.
3. **Preparing ready-to-use *B. tabaci* LAMP kit (protocol for 20 units)**
  1. Use scissors to cut 8-tube LAMP strips into two 4-tube LAMP strips.
  2. Label the tubes of the 4-tube LAMP strips according to the scheme shown in Figure 1.
  3. Prepare *B. tabaci* LAMP reaction mastermix (protocol for 80 reactions).
    1. Add 1195.1 µL of ready-to-use GspSSD isothermal master mix (containing GspSSD polymerase, pyrophosphatase, magnesium sulfate, deoxynucleotides, double strand binding DNA binding dye) and 717.4 µL of *B. tabaci* LAMP primer mix to a 2 mL microcentrifuge tube. Briefly vortex and pulse centrifuge.
    2. Dispense 22.5 µL of *B. tabaci* LAMP reaction mastermix (prepared in step 1.3.3.1) into each tube of the 4-tube LAMP strips (prepared in step 1.3.1) and pulse centrifuge.
  4. Vortex the *B. tabaci* LAMP PAC (prepared in step 1.2) quickly and pulse centrifuge. Then, add 2.5 µL into the tube labelled with “PAC” of each 4-tube LAMP strip (Figure 1).
  5. Close lids and store the ready-to-use *B. tabaci* LAMP kit units at -20 °C. NOTE: Use them within 1 year.

### 2. On-site LAMP Analysis

1. DNA extraction
  1. Use sterile toothpicks to transfer the insect specimens into 0.5 mL microcentrifuge tubes containing 30 µL of DNA extraction solution (prepared in step 1.1.2). NOTE: Make sure that the insects are immersed in the extraction solution.
  2. Incubate the samples for 5 min at 95 °C in a thermomixer (300 rpm). Briefly vortex and pulse centrifuge.
2. *B. tabaci* LAMP assay
  1. Thaw a ready-to-use *B. tabaci* LAMP kit prepared in step 1.3. Vortex quickly and pulse centrifuge. NOTE: With each kit, it is either possible to test two different specimens or to analyze the DNA extract of one specimen in duplicate.
  2. Add 2.5 µL of sample DNA extract (prepared in step 2.1) into the tubes labeled “S1” and “S2” of the ready-to-use *B. tabaci* LAMP kit (Figure 1).
  3. Add 2.5 µL of pure alkaline DNA extraction solution (prepared in section 1.1) into the tube labeled “NAC” for the negative amplification control (Figure 1).
  4. Vortex the ready-to-use *B. tabaci* LAMP kit quickly and pulse centrifuge.
  5. Insert the ready-to-use *B. tabaci* LAMP kits into the LAMP analysis device (with real-time fluorescence measurement) or a real-time PCR platform and perform an isothermal DNA amplification analysis at 65 °C for 60 min.
  6. Measure the melting temperatures of DNA amplification products by heating up to 98 °C with a subsequent cooling step (ramp rate of 0.05 °C/s) to 75 °C, while measuring fluorescence in real-time.
3. LAMP assay read-out
  1. Validate the LAMP read-out manually as follows.
    1. If DNA amplifications were measured for the sample and the PAC, no DNA amplification was measured for the NAC, and the annealing temperature of the amplification products were between 80.0 and 85.5 °C, consider the LAMP results as POSITIVE (Figure 2).
    2. If there is no DNA amplification for the samples (*i.e.,* tubes labeled S1 and S2) but for PAC and NAC then consider the LAMP result as NEGATIVE (Figure 2).
    3. If DNA amplification was measured for the samples, but the annealing temperatures of corresponding amplification products were outside the range 80.0 ‒ 85.5 °C, and/or PAC gave no DNA amplification, and/or NAC gave a DNA amplification, consider the LAMP result as INVALID (Figure 2).
  2. Optionally, validate the LAMP read-out using the LAMP validation application (**Supplemental file 1**).
    1. Define target species and define the number of tested samples. Click the *"Generate Report"* button.
    2. Transfer the read-out (DNA amplification yes/no, annealing temperature amplification product, results of PAC and NAC) from the on-site LAMP analysis device or real-time PCR platform to the corresponding input fields of the validation application. The result of the validation is immediately displayed after entering the data.

## Representative Results

During the validation of the *B. tabaci *LAMP assay, insect specimens intercepted in the course of the regular Swiss import control process were analyzed^8^. The specimens originated from eight different countries (Canary Islands, Dominican Republic, Israel, Malaysia, Morocco, Singapore, Thailand, and Vietnam) and reflect the genetic diversity of *B. tabaci* found at European POEs^8^. All LAMP results were cross-validated by DNA barcoding^8^.

From a total of 80 specimens analyzed by LAMP, 75 specimens (93.8%) were correctly identified as *B. tabaci *(true-positives), two specimens (2.5%) were correctly identified as not being *B. tabaci* (true-negatives), and three specimens (3.8%) were wrongly identified as not being *B. tabaci* (false-negatives) (**Table 2**)^8^. The correct-negative results originated from two *Trialeurodes vaporariorum* specimens, a non-regulated species at high risk to be confused with *B. tabaci* at POEs for plant products^8^. Based on these results, the following measurements of diagnostic accuracy were calculated: test specificity (true-negative rate), 100%; test sensitivity (true-positive rate), 96.2%; test efficiency (percentage of correct test results), 96.3% (**Table 2**)^8^. When assessing the analytical sensitivity (detection limit), the *B. tabaci* LAMP assay successfully amplified sample DNA diluted to 100 fg/µL across three technical replicates (**Table 3**).

A subset of the assays (N = 13) was performed under on-site conditions at the Swiss POE Zurich Airport by plant health inspectors using the ready-to-use *B. tabaci* LAMP kits^8^. When cross-validated in the reference laboratory, all results from on-site testing were found to be correct (test efficiency = 100%)^8^. Assessing the on-site LAMP assay performance, the average time to positive (time until a positive results was available) was 38.4 ± 10.3 min (mean ± standard deviation)^8^. A representative DNA amplification plot and the corresponding annealing derivative from a *B. tabaci* LAMP analysis performed under on-site conditions are shown in Figure 3A** and B**. In this example, sample one and two were correctly identified as *B. tabaci* indicated by DNA amplification after approximately 30 min (Figure 3A) together with the expected annealing temperatures at approximately 82 °C (Figure 3B).

**Figure:**
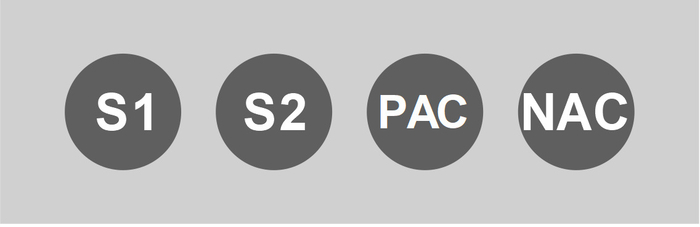

**Figure 1: Visualization of the experimental set-up of a ready-to-use *B. tabaci* LAMP kit described in the protocol. **S1, sample 1; S2, sample 2; PAC, positive amplification control; NAC, negative amplification control. Please click here to view a larger version of this figure.

**Figure:**
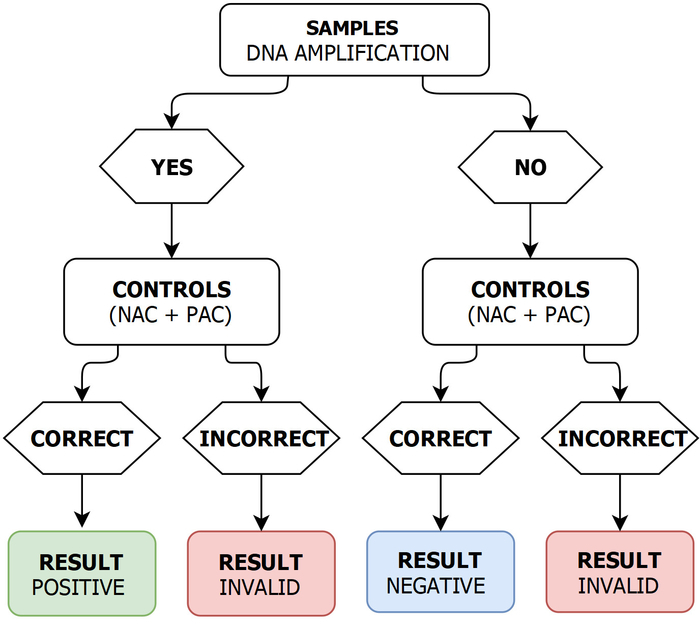

**Figure 2: LAMP read-out validation schema.** PAC: positive amplification control; NAC: negative amplification control. Please click here to view a larger version of this figure.

**Figure:**
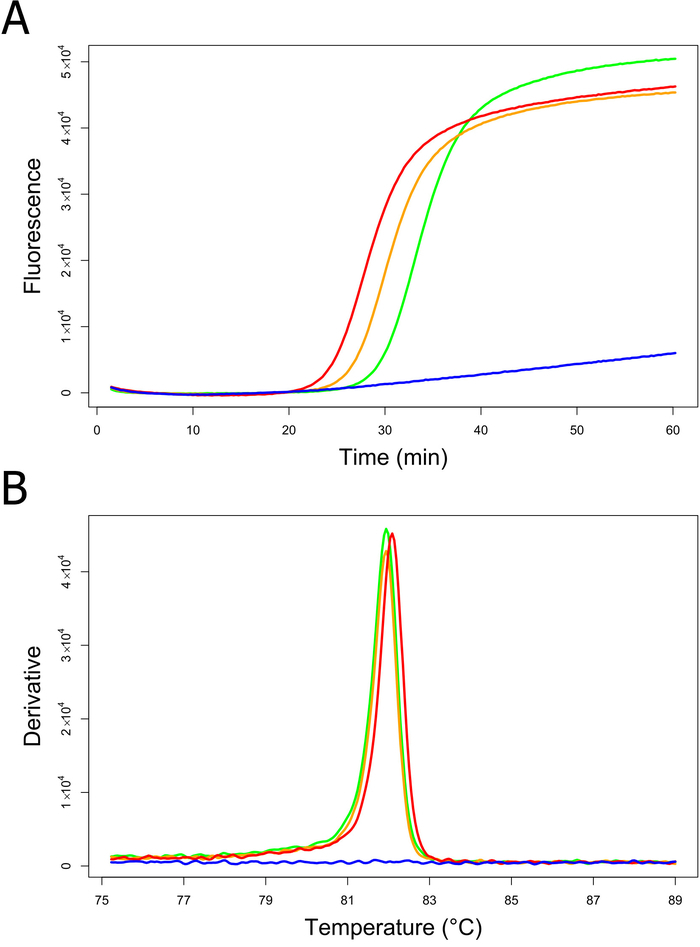

**Figure 3: DNA amplification plot (A) and annealing derivative (B) of a *B. tabaci* LAMP analysis performed under on-site conditions.** Fluorescence was measured in relative intensity units. Green line, sample 1; orange line, sample 2; blue line, negative amplification control (NAC); red line, positive amplification control (PAC). Please click here to view a larger version of this figure.

**Table:** 

**Component**	**Stock conc.**	**Final reaction conc.**	**Volume per reaction**
Taq Polymerase Master Mix	2x	1x	10 µL
Primer C1-J-2195	20 µM	0.4 µM	0.4 µL
Primer TL2-N-3014	20 µM	0.4 µM	0.4 µL
Molecular Grade Water	-	-	8.2 µL
DNA Template	-	-	1 µL

**Table 1: Preparation of PCR reaction mastermix for the *****B. tabaci***** positive amplification control. **Components and concentrations needed to set up one PCR reaction. The final reaction volume is 20 µL. Primer sequences are shown in 1.2.1.1.

**Table:** 

**N**	**N_TP_**	**N_FP_**	**N_TN_**	**N_FN_**	**SEN (%)**	**SPE (%)**	**EFF (%**)
80	75	0	2	3	96.2	100	96.3

**Table 2: Results of the *B.tabaci* LAMP assay validation.** N, number of analyses; N_TP_, number of true-positive results; N_FP_, number of false-positive results; N_TN_, number of true-negative results; N_FN_, number of false-negative results; SEN, diagnostic sensitivity; SPE, diagnostic specificity, EFF, test efficiency. 

**Table:** 

**C_DNA_**(fg/µL)	**N_PR_**	**T_P _**(min) (mean ± SD)	**T_A _**(°C) (mean ± SD)
1 x 10^5^	3	33.5 ± 2.9	81.3 ± 0.1
1 x 10^4^	3	30.7 ± 1.1	81 ± 0.0
1 x 10^3^	3	40.4 ± 3.9	81.1 ± 0.1
1 x 10^2^	3	50.7 ± 1.6	81.1 ±0.1
1 x 10^1^	0	-	-
1 x 10^0^	0	-	-

**Table 3: Analytical sensitivity (detection limit) of the *****B. tabaci***** LAMP assay. **Each dilution was tested in triplicates. C_DNA_, DNA concentration per reaction; N_PR_, number of positive replicates; T_P_, time until a positive result was available; T_A_, annealing temperature; SD, standard deviation.

## Discussion

The ability to accurately identify potentially harmful organisms without time delay represents a critical aspect for the management of pest species^9^,^10^,^26^. Besides being rapid, for plant import products, an ideal pest identification method should be simple to perform on-site at POEs^8^,^26^. This paper reports the protocol of a novel LAMP assay for the rapid identification of *B. tabaci*, a quarantine insect organism frequently intercepted at European borders (https://ec.europa.eu/food/sites/food/files/plant/docs/ph_biosec_europhyt_annual-report _2016.pdf).

The rationale behind the development of the diagnostic test was to design an easy-to-follow protocol which can be performed during the plant import control procedure by plant health inspectors with minimal laboratory training. In order to make on-site testing as rapid and simple as possible, the protocol is divided into two parts, the preparation of a ready-to-use kit and the actual performance of the LAMP assay. The first part may be done in an external laboratory so that the plant health inspector can perform the DNA extraction and LAMP assay on-site with only one pipetting step.

Though only one step, pipetting small amounts of liquid may be challenging for users with little or no laboratory experience. To address this issue, a dye (cresol red) is added to the extraction solution so that the operator can visually confirm the small amount (*i.e.,* 2.5 µL) of DNA is correctly transferred to the respective tube. Another important simplification of the protocol is the validation application as it facilitates a reliable interpretation of the LAMP read-out (**Supplemental file 1**).

The novel *B. tabaci* LAMP assay has been validated under laboratory and on-site conditions by testing insect specimens intercepted during the regular import control process of Switzerland^8^. In total, 80 specimens from three continents, Africa, Eurasia, and North America, were analyzed by LAMP. Of the 80 specimens, only three (3.8%) were wrongly identified (false-negatives)^8^. When analyzing the primer target DNA sequences of the false-negative specimens, it was found that they were new *B. tabaci* haplotypes that have so far not been described^8^. Based on these results, the *B. tabaci* LAMP primer set has been modified and successfully re-validated^8^.

One major limitation of any DNA amplification-based method including LAMP is that they only identify pre-defined target DNA sequences^8^,^27^. A comprehensive knowledge of the genetic variation found in the primer target sequence is therefore crucial to ensure diagnostic accuracy^8^,^27^. However, such information is often very limited, especially in the case of newly emerging pest species^8^. Though rare, false-negative results caused by mutations in the target sequence are expected^8^. In the case of the present *B. tabaci* LAMP assay, a solution for this problem is the combination with a DNA barcoding-based technology, a strategy realized in the course of the implementation of this diagnostic test at the POE Zurich Airport^8^. Here, all LAMP-negative results were re-analyzed by DNA barcoding in an external laboratory^8^. In case a novel pest haplotype not yet described is encountered, the LAMP primers can be modified using the DNA sequence generated in the barcoding process^8^. Thereby, the resulting loss of speed in case of a negative LAMP result is compensated for the maximum diagnostic accuracy ensured in this two-stage process^8^.

The set-up costs for the current LAMP assay at a POE are approximately USD 25,000. With the increasing number of LAMP tests developed for plant pests (*e.g., Erwinia amylovora*, Flavescence dorée, and *Guignardia citricarpa*), such a one-time investment appears justified^13^,^15^,^28^. However, the protocol could potentially be modified to reduce these costs even further. For example, for the DNA extraction step at 95 °C the thermo mixer used here could be replaced by a less expensive water bath, or by performing this step directly in the real time LAMP device. Furthermore, the mixing steps on the vortex could probably be replaced by manually flicking the tubes, and in the DNA transfer step the pipettor might be replaced by sterile inoculation loops.

Future improvements for a rapid identification of *B. tabaci* and pest species in general could be an implementation of an on-site sequencing approach that would allow to perform DNA barcoding analyses at POEs. A promising candidate system for such an implementation is the nanopore sequencing technology. Indeed, the technology has recently been successfully implemented in an on-site DNA barcoding effort to assess the biodiversity of a rainforest^8^,^29^,^30^. An on-site DNA barcoding identification system can completely replace the need for the development of targeted diagnostic tests and their validation. Also it allows collecting additional information about pest characteristics such as pesticide resistance genes^8^. Nevertheless, until novel sequencing technologies will be implemented routinely, the *B. tabaci* LAMP assay represents a rapid (<1 h) and accurate identification method.

## Disclosures

The author Michael Andreou is a shareholder of OptiGene Limited that produces reagents and instruments used in this article. The other authors have nothing to disclose.
